# Evaluation of the Lodestar DX: a comparison with traditional culture for the detection of bacteriuria in the workup of urinary tract infection

**DOI:** 10.1093/jacamr/dlaf128

**Published:** 2025-07-22

**Authors:** Stuart Drazich-Taylor, James Moore, Brian McCann

**Affiliations:** Department of Biological Sciences, University of East Anglia, Research Park, Norwich, Norfolk NR4 7TJ, UK; Department of Microbiology, Norfolk and Norwich University Hospital, Colney Lane, Norwich, Norfolk NR4 7UY, UK; Department of Microbiology, Norfolk and Norwich University Hospital, Colney Lane, Norwich, Norfolk NR4 7UY, UK; Department of Microbiology, Norfolk and Norwich University Hospital, Colney Lane, Norwich, Norfolk NR4 7UY, UK

## Abstract

**Background:**

The Lodestar DX is a point-of-care test (POCT) using loop-mediated isothermal amplification (LAMP) to detect common uropathogens. Novel POCTs may provide an adjunct in the diagnostic work up of urinary tract infection (UTI).

**Objectives:**

To evaluate the performance of the Lodestar DX in the identification of bacteriuria.

**Methods:**

We compared the Lodestar DX against urine culture results from August to November 2024 at the Eastern Pathology Alliance in non-pregnant adults.

**Results:**

379 urine samples were tested on the Lodestar DX. 344 (90%) of these passed both internal controls. Summary Lodestar DX performance was sensitivity 85.6% (95% CI 69.2%–94.6%), specificity 92.0% (95% CI 88.1%–94.9%), positive predictive value 64.1% (95% CI 48.7%–77.2%), negative predictive value 97.0% (95% CI 94.0%–98.6%) and overall accuracy 91.2% (95% CI 87.5%–94.0%).

In cultures positive for their respective organisms, the sensitivity was 87.9% (*n* = 66, 95% CI 77.5%–94.6%) for *Escherichia coli*, 80% (*n* = 20, 95% CI 56.3%–94.3%) for *Enterococcus*, 27.3% (*n* = 11, 95% CI 6.0%–61%) for *Staphylococcus aureus*, 92.3% (*n* = 13, 95% CI 64.0%–99.8%) for *Staphylococcus saprophyticus*, 93.8% (*n* = 16, 95% CI 69.8%–99.8%) for *Proteus mirabilis*, 84.2% (*n* = 19, 95% CI 60.4%–96.6%) for *Pseudomonas aeruginosa* and 100% sensitivity (*n* = 2, 95% CI 15.8%–100%) for *Klebsiella pneumoniae*.

**Conclusions:**

In summary, the Lodestar DX had good performance for the detection of uropathogens apart from *S. aureus*. Further comparison with other POCTs would be beneficial.

## Introduction

Urinary tract infections (UTIs) are among the most common bacterial infections globally, occurring in both healthcare and community settings.^1^ In the UK, UTIs accounted for 22% of all antibiotic prescriptions in primary care during 2019–2020, making them the second most common reason for antimicrobial prescriptions.^2,3^ Inappropriate antimicrobial prescribing is a major contributor to antimicrobial resistance, a recognized global health emergency and a growing cause of morbidity and mortality worldwide.^4,5^ Addressing this challenge, the UK Government has outlined an ambitious antimicrobial resistance action plan, including a target to reduce total antibiotic use in humans by 5% from 2019 levels over the subsequent decade.^6^ Reducing prescribing for UTIs is a key component.

The diagnosis of a UTI is based on clinical symptoms, with empirical antibiotic treatment initiated while awaiting microbiological confirmation. A positive culture may lead to antibiotic rationalization but will take 24–48 hours for a provisional result. Rapid diagnostic methods such as dipstick tests can detect markers associated with bacterial infections, including leukocyte esterase and nitrites. However, these tests have limited diagnostic accuracy, and are not recommended for catheterized patients or individuals >65 years of age, due to their low positive predictive value (PPV).^7^ The reduced PPV in older adults is partially attributable to the higher prevalence of Gram-positive organisms, which do not produce nitrites, and the frequent occurrence of asymptomatic bacteriuria (ASB).^8^ Colonization by non-pathogenic strains may offer protection against more virulent pathogens, and studies have shown that treating ASB can increase the short-term risk of developing pyelonephritis.^9,10^

The National Institute for Health and Care Excellence (NICE) has emphasized the need for advancements in tools to support the diagnostic pathway of UTIs, with particular interest in novel point-of-care tests (POCTs).^11^ The private biotechnology sector has invested significantly in the development of innovative urine-based POCTs, with some systems already licensed for limited use in the UK.^12^ However, a 2023 systematic review of UTI POCT by Tomlinson *et al.* concluded that current evidence does not yet support their clinical use, but frequent re-evaluation is warranted.^13^ In its 2023 health technology assessment, NICE did not recommend rapid urine POCTs for routine use within the NHS, citing insufficient data on their accuracy and clinical utility. However, NICE strongly advocated for further research with an emphasis on evaluating test accuracy, to determine whether future recommendations could change.^11^

The Lodestar DX diagnostic platform, developed by Llusern Scientific Limited, is a rapid POCT designed to detect the presence or absence of bacteria in urine.^14^ It has recently acquired ISO 13485 certification for recognition of the manufacturer’s commitment to quality assurance.^15^ It employs loop-mediated isothermal amplification (LAMP), a molecular technique that amplifies nucleic acids under isothermal conditions (60–65°C), delivering results within 35 minutes. The LSL-HUTI panel can identify seven common uropathogens on the Lodestar DX platform: *Escherichia coli* (*E. coli*), *Klebsiella pneumoniae* (*K. pneumoniae*), *Proteus mirabilis* (*P. mirabilis*), *Pseudomonas aeruginosa* (*P. aeruginosa*), *Staphylococcus saprophyticus* (*S. saprophyticus*) and *Enterococcus* species/*Staphylococcus aureus* (*S. aureus*) as a combined target, alongside positive and negative controls.

To date, the only published clinical performance evaluation of the Lodestar DX platform has focused on symptomatic adult women suspected of having a UTI. The study demonstrated strong concordance with conventional laboratory detection methods, reporting an overall accuracy of 86.1%.^14^ The present study aimed to evaluate Lodestar DX’s performance in a broader patient population with direct comparison with conventional laboratory methods.

## Aims

To assess the clinical performance of the Lodestar DX diagnostic platform in comparison to standard culture-based methods for the identification of organisms associated with UTIs.

## Methods

Urine samples processed at the Eastern Pathology Alliance between August and November 2024 were considered for inclusion in this study. Samples from individuals <18 years of age and those from antenatal clinics were excluded to minimize samples sent for screening rather than suspected infection. Participant data were retrieved from the Telepath Laboratory Information Management System (LIMS) (DXC Technology Services, Virginia, USA).

Urine samples were processed following standard laboratory protocols. This included fluorescence flow cytometry using the Sysmex UF-5000, followed by inoculation onto orientation agar (BD BBL CHROMagar). In accordance with standard operating procedures (SOPs) samples with white blood cell (WBC) counts <35/µL and/or bacterial counts ≤9000/µL on flow cytometry, which did not meet at-risk criteria, were excluded from culture. A report is issued indicating that findings were not suggestive of infection. Risk criteria for automatic culture included samples from delivery suites, haematology/oncology wards, and intensive care units. Culture is performed by manual inoculation of 2 µL of urine onto BD BBL CHROMagar and spreading by the calibrated loop method onto a quarter plate as per laboratory SOPs. This was incubated at 35–37°C for 18–24 hours before being read and the results recorded. Disc sensitivities were performed on Muller–Hinton agar based on EUCAST guidance.

**Figure 1.**
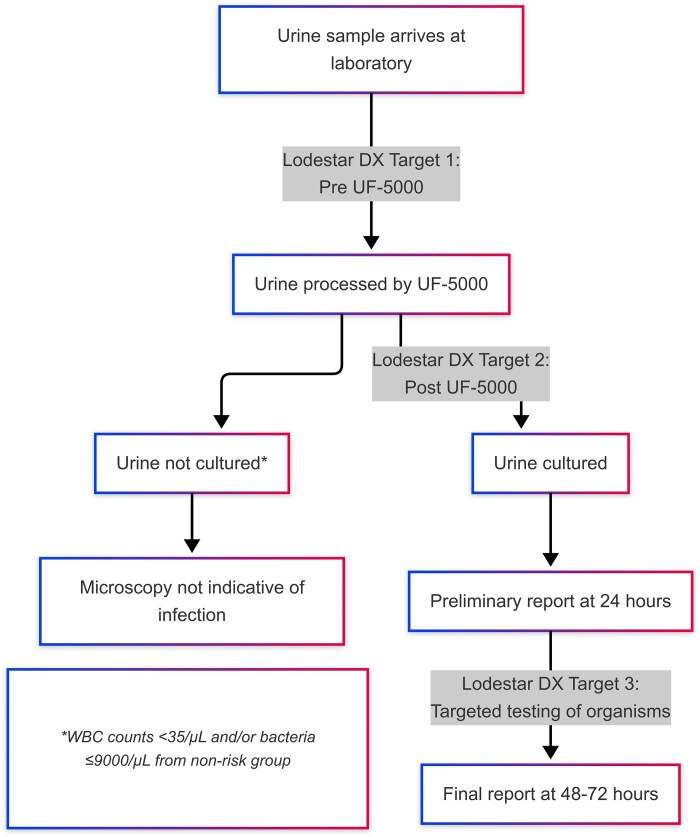
Flowchart of urine processing and Lodestar DX testing.

To ensure a representative sample set, three categories of urine specimens were tested on the Lodestar DX: (i) samples arriving at the laboratory prior to flow cytometry, (ii) samples flagged as positive during flow cytometry and (iii) targeted testing of organisms included in the LSL-HUTI panel identified on preliminary culture (Figure 1).

For testing on the Lodestar DX platform, a 10-µL urine sample was mixed with a diluent, and 5 µL of the resulting mixture was added to the first seven reaction wells of the connected eight-well cassette, with the final well serving as the negative control. The cassette lids were sealed, and the cassette was gently inverted before being placed into the open Lodestar DX device. Once the lid was closed, labelled channels corresponding to the eight wells became visible, each with a red and green light.

The first six channels represent bacterial targets, followed by the positive and negative controls. A channel was considered positive if the red light was illuminated, and negative if the green light was illuminated at the completion of the test run. If the controls passed, the test panel was recorded, the cassette discarded and the Lodestar DX was prepared for the next sample.

Samples that failed the positive control were diluted 1:2 and retested in accordance with the manufacturer’s recommendations. All testing procedures were performed by trained investigators following the manufacturer’s instructions.

**Figure 2.**
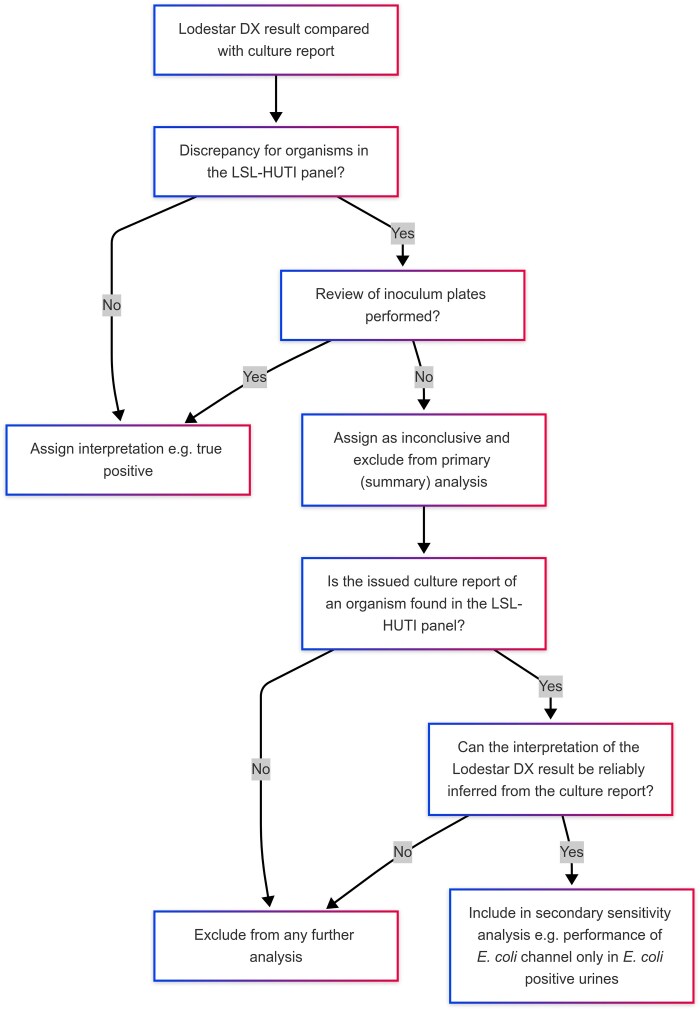
Process for review of inoculum plates and assignment of interpretation.

The primary (summary) analysis was the concordance of results between the Lodestar DX and standard culture methods. In cases of uncertainty or discrepancies between the two methods, the original orientation agar inoculum was reviewed. (Figure 2) This was usually performed within 72–96 hours of the urine sample being received in the laboratory. If growth consistent with an organism identified by the Lodestar DX was observed, the result was classified as a true positive. Conversely, missed detections were classified as false negatives. Discrepant samples that were not rereviewed due to plate discardment were not included in the primary analysis. However, they could be eligible for secondary sensitivity analysis of Lodestar DX performance in cultures positive for organisms included in the LSL-HUTI panel.

Data was analysed in R studio build 467. The Mann–Whitney *U*-test was used to compare the distribution between non-paired samples. Confidence intervals were calculated using the Clopper–Pearson method. Correlation between continuous and ordinal variables were calculated using the biserial correlation method and between ordinal variables, Spearman’s rank was used.

## Results

### Control failures

In total, 379 urine samples were tested on the Lodestar DX, and 344 samples (91%) passed internal quality checking and did not have any control failures (Table 1). Of the 35 that failed an internal control, 31 had isolated positive control failures and there were 3 negative control failures. One sample failed both positive and negative controls. Of the 22 samples that failed internal controls and were retested, 17 passed on repeat testing (77.2%).

**Table 1.** Samples and Lodestar DX controls result

Characteristic	*N* = 379
Control failure, *n* (%)	35 (9.2%)
Positive control failure, *n* (%)	31 (8.2%)
Negative control failure, *n* (%)	3 (0.8%)
Negative and positive control failure, *n* (%)	1 (0.3%)
Failed samples that were retested, *n* (%)	22/35 (62.9%)
Passed both controls on retesting, *n* (%)	17/22 (77.2%)

**Figure 3.**
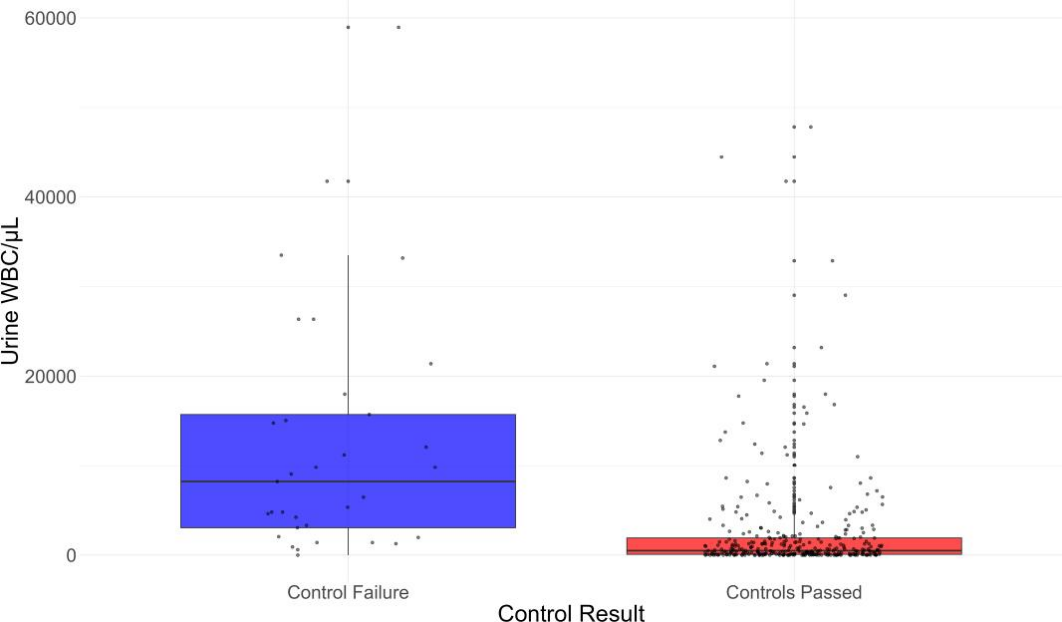
Urine WBC count plotted against Lodestar DX control result.

Figure 3 shows the data for the 372 samples that had urine WBC count (WBC/µL) recorded in relation to control failures on the Lodestar DX. The median WBC for samples that failed on the Lodestar DX was 8213 (IQR 3053–15722) in comparison with a median of 498 for samples that passed internal controls (IQR 86.5–1916.5). The difference between these were significant when interrogated by the Mann–Whitney *U*-test (W = 9474, *P* < 0.001).

### Baseline demographics

A total of 337 patients accounted for the 344 samples that passed internal controls. As seen in Table 2 most patients tested were of an older age (mean 68 ± 18 SD), more females (58%, *n* = 196) were tested than males (42%, *n* = 141). Samples from mid-stream urines were most common (83%, *n* = 286) with some catheter urines sampled (17%, *n* = 57). One sample was from a suprapubic aspirate. Urine samples sent from general practitioners (GPs) accounted for 63% of samples (*n* = 215). The remaining 38% (*n* = 129) were from hospitals.

**Table 2.** Demographics of patients and samples that passed Lodestar DX controls

Characteristic	*N* = 344 samples
Age, mean (SD) (337 patients)	68 (18)
Sex, *n* (%) (337 patients)	
* Female*	196 (58%)
* Male*	141 (42%)
Sample type, *n* (%)	
* CSU*	57 (17%)
* MSU*	286 (83%)
* Suprapubic aspirate*	1 (0.3%)
Setting, *n* (%)	
General practice	215 (63%)
Hospital	129 (38%)
Urine WBC count/µL, mean (SD)	2554 (5956)
Time to testing, mean hours (SD)	31 (20)
All channels negative on Lodestar DX, *n* (%)	89 (26%)
More than one positive result on Lodestar DX, *n* (%)	98 (28%)
Samples with three channels positive or more, *n* (%)	34 (9.9%)
Successfully completed plate reviews, *n* (%)	147/190 (77%)
Samples eligible for primary analysis, *n* (%)	301/344 (88%)

Altogether, 89 samples were negative across all channels on the Lodestar DX (26%); and 98 urine samples had more than one positive result on the Lodestar DX (28%). A total of 34 urine samples (9.9%) had three or more positive channels on the Lodestar DX. There was no significant correlation between age (*R* = −0.046, *P* = 0.400) nor sample type (*R* = 0.076, *P* = 0.160) with multiple positives on the Lodestar DX. The mean urine WBC on flow cytometry was 2550/µL (SD 5948). The mean time to testing from sample collection was 31 hours (SD 20). Of the 190 samples requiring plate reviews, 147 were successfully performed (77%) this resulted in 301 of the 344 samples (88%) being eligible for inclusion in the primary analysis.

### Culture reports

**Figure 4.**
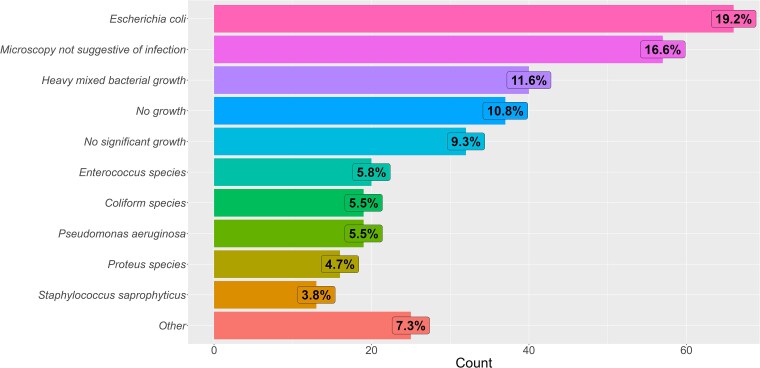
Issued laboratory reports for urine samples passing Lodestar DX controls.

The most frequent culture report was isolation of *E. coli* in 66 samples (19.2%), followed by a report of microscopy not suggestive of infection in 57 cultures (16.6%) (Figure 4). Heavy mixed bacterial growth accounted for 40 samples tested (11.6%). Twenty samples (5.8%) grew an *Enterococcus* species. Undifferentiated coliform species [members of *Klebsiella* spp., *Enterobacter* spp., *Serratia marcescens* and *Citrobacter* spp. (KESC) group] and *P. aeruginosa* were both cultured in 19 samples each (5.5%). Sixteen samples grew a *Proteus* species (4.7%). Thirteen samples (3.8%) grew *S. saprophyticus* with 11 samples growing *S. aureus* (3.2%).

### Primary analysis: overall performance of Lodestar DX

Table 3 shows the performance of the Lodestar DX across the 301 samples eligible for inclusion in the primary analysis and incorporating rereview of initial inoculum. Owing to members of the KESC group only being identified to species level if they are resistant to first line antibiotics, this limited the interpretability of the *K. pneumoniae* results across all samples.

**Table 3.** Interpretation of Lodestar DX performance in all urine cultures

Organism	Sensitivity % (95% CI)	Specificity % (95% CI)	PPV % (95% CI)	NPV % (95% CI)	Accuracy % (95% CI)
*E. coli*	84.7 (75.3–91.6)	87.5 (82.3–91.6)	72.7 (62.9–81.2)	93.6 (89.2–96.5)	86.7 (82.3–90.3)
*Enterococcus/S. aureus*	79.7 (67.2–89.0)	88.8 (84.2–92.5)	63.5 (51.5–74.4)	94.7 (90.9–97.2)	87.0 (82.7–90.6)
*P. aeruginosa*	85.7 (67.3–96.0)	90.8 (86.8–94.0)	49.0 (34.4–63.7)	98.4 (96.0–99.6)	90.4 (86.5–93.5)
*P. mirabilis*	88.9 (70.8–97.6)	95.3 (92.0–97.4)	64.9 (47.5–79.8)	98.9 (96.7–99.8)	94.7 (91.5–96.9)
*S. saprophyticus*	88.9 (65.3–98.6)	97.5 (95.0–99.0)	69.6 (47.1–86.8)	99.3 (97.4–99.9)	97.0 (94.4–98.6)

An averaged summary of the Lodestar performance excluding *K. pneumoniae* would therefore be of a sensitivity of 85.6% (95% CI 69.2%–94.6%), specificity 92.0% (95% CI 88.1%–94.9%), PPV 64.1% (95% CI 48.7%–77.2%), negative predictive value 97.0% (95% CI 94.0%–98.6%) and overall accuracy 91.2% (95% CI 87.5%–94.0%).

### Secondary analysis: performance in culture positive cases

We also reviewed the performance of each Lodestar DX target in urine cultures positive for that organism (Table 4). This was to ascertain the sensitivity for detecting a probably true bacteriuria with the target organism. For example, reviewing the performance of the *E. coli* channel solely in cultures positive for *E. coli.*

**Table 4.** Lodestar DX sensitivity in cultures positive for their respective organisms

Test	Sensitivity % (95% CI)
*E. coli* (*n* = 66)	87.9 (77.5–94.6)
*Enterococcus* (*n* = 20)	80.0 (56.3–94.3)
*S. aureus* (*n* = 11)	27.3 (6.0–61.0)
*S. saprophyticus* (*n* = 13)	92.3 (64.0–99.8)
*P. mirabilis* (*n* = 16)	93.8 (69.8–99.8)
*P. aeruginosa* (*n* = 19)	84.2 (60.4–96.6)
*K. pneumoniae* (*n* = 2)	100.0 (15.8–100.0)

In doing this the sensitivity of *E. coli* rose from 84.7% to 87.9% (*n* = 66, 95% CI 77.5%–94.6%). The sensitivity of *Enterococcus* was 80% (*n* = 20, 95% CI 56.3%–94.3%) while individual *S. aureus* performance had a sensitivity of 27.3% (*n* = 11, 95% CI 6.0%–61%). The sensitivity of *S. saprophyticus* rose from 88.9% to 92.3% (*n* = 13, 95% CI 64.0%–99.8%). *P. mirabilis* sensitivity rose from 88.9% to 93.8% (*n* = 16, 95% CI 69.8%–99.8%). The sensitivity of *P. aeruginosa* was 84.2% (*n* = 19, 95% CI 60.4%–96.6%) down from 85.7% with 100% sensitivity (*n* = 2, 95% CI 15.8%–100%) in the two confirmed *K. pneumoniae* isolates.

## Discussion

Our study shows that the Lodestar DX could be a viable addition to microbiology diagnostics. The overall aggregated performance of the Lodestar DX was good with sensitivity 85.6% (95% CI 69.2%–94.6%), specificity 92.0% (95% CI 88.1%–94.9%), PPV 64.1% (95% CI 48.7%–77.2%), negative predictive value 97.0% (95% CI 94.0%–98.6%) and overall accuracy 91.2% (95% CI 87.5%–94.0%). A potential limitation of the assay is the low PPV and frequency of multiple positive channels (28%). This may represent a contaminated sample rather than infection by multiple organisms and there is a concern that this could lead to increased antimicrobial prescribing or consultations to microbiology for interpretation of results. The low PPV could have been influenced by previous antibiotics to sampling, limiting growth of sensitive organisms, unfortunately data concerning this was not available for review.

Although urinalysis and culture can aid in supporting a diagnosis of UTI, clinical assessment should remain the cornerstone of diagnosis. Diagnostic challenges may arise in cases presenting with non-specific symptoms or in patient populations with limited ability to communicate effectively. Perhaps, in these cases the Lodestar DX’s greatest utility would be in its NPV guiding a decision on whether to commence empirical treatment, rather than linking antimicrobial cover to the specific organisms identified. If the Lodestar DX was negative, holding off empirical treatment and exploring alternative diagnoses could be considered. There would need to be cognisance however that certain *Enterobacterales* such as *Enterobacter* spp. are not currently included within the LSL-HUTI panel. Local data collected within our region have shown that these organisms may account for ∼2% of positive urine cultures with *Enterobacterales*.^16^

The Lodestar DX may have an advantage over the urine dipstick in the detection of Gram-positive organisms such as *S. saprophyticus*, which are not nitrite producers. The presence of leukocyte esterase in urine is relatively non-specific and by itself is not considered a reliable indicator for a UTI.^17^ The detection of bacteriuria in the absence of clinical criteria for UTI, as would be expected if the Lodestar DX was used in asymptomatic patients, raises concerns it could lead to misdiagnosis and inappropriate antimicrobial prescribing. However, in highly specific settings where it may be appropriate to screen for ASB such as in intermediate or high risk pregnancies, or before certain urological procedures where the mucosal barrier is expected to be breached, it may have utility.^18,19^

Most UTIs are caused by *E. coli* and any novel POCT must be powered to detect *E. coli*.^20^ The sensitivity and specificity of the Lodestar DX in *E. coli* detection was 84.7% (95% CI 75.3%–91.6%) and 87.5% (95% CI 82.3%–91.6%), respectively, with an overall accuracy of 86.7% (95% CI 82.3%–90.3%). When looking at sensitivity in urine cultures that grew *E. coli* this rose to 87.9% (*n* = 66, 95% CI 77.5%–94.6%). This was lower than reported in a previous performance evaluation of the Lodestar DX although our study included a broader patient population.^14^ Overall performance of the Lodestar DX was equally strong *in S. saprophyticus*, *P. mirabilis* and *P. aeruginosa* with sensitivities all >85%. In the two confirmed *K. pneumoniae* isolates the sensitivity was 100%.

The performance of the *Enterococcus*/*S. aureus* channel overall was weaker with a sensitivity of 79.7% (95% CI 67.2%–89.0%). On further analysis this was due to poor *S. aureus* sensitivity of 27.3% (*n* = 11, 95% CI 6.0%–61%), with *Enterococcus* performance in *Enterococcus* positive cultures having a sensitivity of 80% (*n* = 20, 95% CI 56.3%–94.3%). On discussion with the manufacturers, *S. aureus*’ inclusion in the channel is a consequence of sequence homology in the target gene, rather than an intended target. Fortunately, *Staphylococcus aureus* is an uncommon cause of UTIs, and its poor sensitivity may have limited consequences for most patients. However, on rare occasions, the isolation of *S. aureus* in urine can indicate an underlying disseminated infection, where early identification through urinalysis may contribute to improved patient outcomes.^21^ It is possible that the weak performance in this channel could cause delayed recognition or false reassurance.

Furthermore, there was a degree of overlap between the *Enterococcus*/*S. aureus* and *S. saprophyticus* results. In 7/13 (54%) of *S. saprophyticus* positive cultures the *Enterococcus*/*S. aureus* channel was also positive. This may also represent further sequence homology between staphylococcal species. *S. saprophyticus* is a common cause of UTIs in young women^22^ and *Enterococcus* unusual, and therefore the correct interpretation of both channels being positive would likely be of a *S. saprophyticus* UTI in the appropriate clinical context. Further work is necessary to ascertain the degree of overlap between the *Enterococcus*/*S. aureus* and *S. saprophyticus* channels.

Despite the potential applications, there remain barriers to use of the Lodestar DX as a POCT test. Of the isolates tested, 9% failed on internal quality control measures, however, 77% of these worked on retest (with 1:2 dilution if positive control failure). Urines that failed internal controls had a higher median WBC than those that passed, which is consistent with manufacturer guidance that more turbid urines may be more likely to fail. Whether turbid urines should be diluted 1:2 for the first test is not clear. Furthermore, knowing the organisms present within a urine sample without antibiotic sensitivities may limit the tests’ applicability, but can be mitigated by guidelines incorporating local antibiogram data. The requirement for pipetting and cold-chain storage also complicates the logistics of testing, rendering it impractical for near-patient use in its current iteration. There is also a need to develop appropriate middleware for integration with LIMS or electronic patient records. Steps to address these technical factors by the manufacturer are underway and could result in a product suitable for use within GP practices or hospital wards. These changes would be welcomed and facilitate further trials to assess the impact of the Lodestar DX in clinical settings.

There has also been development of several other rapid urine POCTs such as the Astrego PA100-AST (Sysmex Astrego), which provides antibiotic sensitivity data, and the Uriscreen (Savyon Diagnostics), which can detect bacterial catalase in 2 minutes. The Lodestar DX and other novel urine POCT systems are being compared with conventional methods in a GP setting within the TOUCAN study; its results are highly anticipated.^23^ An additional interesting comparison would involve evaluating patient outcomes in low-resource settings where culture facilities are unavailable, specifically comparing cases tested using the Lodestar DX platform to those managed empirically without urinalysis.

Our study had several strengths. Using a single laboratory ensured standardization of urine processing improving reliability of our data. We also rereviewed the initial inoculum on orientation agar to give additional context to the Lodestar DX results. However, agar plates after completion of culture were kept within the laboratory at room temperature. Our overall analysis therefore may have been influenced by relatively trivial growth on the inoculum agar which could have occurred with prolonged culture. A limitation of the study’s design, which involved the random selection of specimens, was the inability to validate whether urine sampling was clinically appropriate or if proper sampling techniques were followed. While this limitation prevents confidently attributing isolated bacteria to a true UTI, it would not be expected to affect the assay’s performance in detecting bacteriuria within a given sample.

In summary, although the authors consider this technology promising and have discussed potential applications, the specific clinical settings in which it could serve as an adjunct in the diagnostic workup of suspected UTIs have yet to be clearly defined. Targeted refinements aimed at improving user-friendliness and reducing operational complexity could further enhance the practical utility of the Lodestar DX platform. However, additional research, particularly in near-patient clinical settings, is necessary to define its precise scope of application. Specifically, its ability to identify bacterial species without providing sensitivity data must be evaluated to determine whether it can have a role in influencing prescribing decisions. Furthermore, comparative studies are needed to evaluate whether it offers advantages over other rapid urine POCT devices in development.
